# Effects of Lactic Acid Bacteria on Reducing the Formation of Biogenic Amines and Improving the Formation of Antioxidant Compounds in Traditional African Sourdough Flatbread Fermentation

**DOI:** 10.3390/antiox13070844

**Published:** 2024-07-14

**Authors:** Alaa Ahmed Alsiddig Hassan, Young Hun Jin, Jae-Hyung Mah

**Affiliations:** Department of Food and Biotechnology, Korea University, Sejong 30019, Republic of Korea; alaa16@korea.ac.kr (A.A.A.H.); younghoonjin3090@korea.ac.kr (Y.H.J.)

**Keywords:** fermented sorghum-based foods, kisra, biogenic amines, tyramine, histamine, antioxidant compounds, total phenolic content, fermentation, lactic acid bacteria, *Sorghum bicolor* (L.) Monesh

## Abstract

This study investigated the safety and functionality of traditional African sourdough flatbread (kisra), based on the content of biogenic amines (BAs) and antioxidant compounds and their improvement using lactic acid bacteria (LAB) species. The primary BAs detected in naturally fermented kisra were tyramine, histamine, putrescine, and cadaverine, with putrescine being the most abundant after baking. In vitro BA production of microorganisms isolated from kisra sourdough revealed that the *Enterococcus* genus contributed to tyramine accumulation, whereas presumptive yeasts may contribute to putrescine and cadaverine accumulation. The use of LAB species, including *Lactiplantibacillus plantarum*, *Limosilactobacillus fermentum*, *Levilactobacillus brevis*, and *Weissella cibaria*, significantly reduced putrescine content to less than about 23% of that of naturally fermented kisra, and eliminated tyramine, histamine, and cadaverine formation. Meanwhile, DPPH scavenging activity, total polyphenolic content, and tannin content in naturally fermented kisra were 85.16%, 1386.50 µg/g, and 33.16 µg/g, respectively. The use of LAB species did not affect the DPPH scavenging activity or tannin content but significantly increased the total phenolic content by up to 20% compared to naturally fermented kisra. Therefore, fermentation with LAB starter cultures might be a promising approach to improve the safety related to BAs as well as the functionality of kisra bread.

## 1. Introduction

*Sorghum bicolor* (L.) Monesh, which is commonly known as sorghum, is the fifth most important grain crop in the world in terms of cultivated area and amount. The largest producing countries are the USA, followed by Nigeria, India, Mexico, Ethiopia, and Sudan (FAOSTAT, 2021) [1], while the African continent accounts for the largest share (>65%) of global sorghum production area. In Africa, sorghum grain is the second-most important grain crop after maize [2]. In Sudan, several indigenous fermented foods are prepared based on the natural fermentation of sorghum [3], which include fermented flatbread (kisra), and thick porridge (aceda), along with alcoholic (marisa) and nonalcoholic (hulu-mur) beverages [4]. Kisra is a traditional lactic-acid-fermented leavened flat pancake-like bread primarily prepared using whole sorghum flour and is a staple food consumed in Sudan [3,5]. Kisra serves as the main dish of the typical Sudanese meal and is generally eaten with meat and vegetable stews (mullah) [5]. It is produced by back-slopping using spontaneous fermented sourdough locally known as “ajin”. In detail, to prepare kisra, a small amount of prefermented “ajin” dough is added to a liquid paste of sorghum flour and water mixed in a ratio of approximately 1:2 and left for 12–18 h to ferment under natural conditions at an optimum temperature of 35–38 °C [6]. Next, the baking process termed “awasa” is performed on hot steel plates (150–160 °C) of various sizes [6]. Regarding the microbial communities involved in the natural fermentation of kisra, Ali and Mustafa [7] found that the predominant microorganisms in fermented kisra dough were *Limosilactobacillus fermentum* and *Lactobacillus amylovorus*. Mohammed et al. [8] also reported that the bacterial population during 24 h of kisra fermentation consisted of *Levilactobacillus brevis*, *Lactobacilli* spp., *Pediococcus pentosaceus*, and *Enterococcus faecium*. Among them, *P. pentosaceus* was the dominant microorganism at the end of the 24 h fermentation. However, Hamad et al. [9] reported that *E. faecalis*, *Lactococcus lactis*, and *L. fermentum* were dominant in the middle stage of sorghum sourdough spontaneous fermentation. Meanwhile, several previous studies have also documented the involvement of yeast in the fermentation process of kisra, with *Saccharomyces cerevisiae* [7], *Candida krusei* [10], *C. intermedia*, and *Debaryomyces hansenii* [8] being detected in fermented kisra sourdough.

The fact that most indigenous foods, including kisra, are produced on a household scale, depending on natural fermentation, parallels issues of inconsistent quality, hygienic risks, and short shelf life [11]. Despite the numerous advantages of consuming fermented foods, the major concerns related to these foods are food safety issues, particularly the occurrence of biogenic amines (BAs). BAs are nonvolatile low-molecular-weight nitrogenous organic bases derived through the decarboxylation of corresponding amino acids by substrate-specific enzymes of decarboxylase-positive microorganisms [12]. BAs have potential hazards when they exceed the safety limits and are of concern in relation to both food spoilage and food safety. Hence, in Europe, commission regulation (EC) No. 2073/2005 set a limit of 100 mg/kg for histamine in fish products [13]. The Australian and New Zealand Food Standards Code states that the level of histamine in fish or fish products must not exceed 200 mg/kg [14]. Marcocbal et al. [15] reported that for healthy individuals, a dietary intake of tyramine in the range of 200–800 mg/kg or more causes a slight increase in blood pressure, whereas concentrations as low as 6 mg/kg may be potentially toxic to patients undergoing treatment with monoamine oxidase (MAO) inhibitors. Currently, there is no regulatory limit for putrescine in food, but toxicological data set the acute oral toxicity in rats at 2000 mg/kg body weight. Rauscher-Gabernig et al. [16] also suggested maximum endurable concentrations of putrescine to be 140 mg/kg in fermented cabbage, 180 mg/kg in cheeses, and 510 mg/kg in seasoning products.

The detection of BAs, including histamine, putrescine, cadaverine, and tyramine, in African fermented sorghum-based foods has been specifically confined to sorghum beverages such as sorghum beer and related products [17,18,19]. However, BAs have not yet been investigated in kisra bread or any fermented sorghum-based foods. Considering that kisra is a lactic-acid-fermented food rich in protein and free amino acids [20] that can be used by decarboxylase-positive lactic acid bacteria (LAB) abundant in kisra sourdough, it is necessary to monitor the formation of various BAs. Therefore, this study was conducted to investigate the formation of BAs during the natural fermentation of kisra bread and also evaluate the potential of LAB species commonly selected as commercial starters to inhibit BA accumulation during kisra fermentation. Since naturally fermented kisra bread contains antioxidant compounds [21], the study further assessed the contribution of the LAB species to the functionality of kisra bread by analyzing changes in antioxidant compounds and activity during fermentation. This study can expand the knowledge on the formation of BAs and antioxidant compounds in sorghum-based fermented foods, particularly kisra bread, and provide selection criteria at the species level for lactic acid bacteria strains that can be used in the industrial production of traditional African sourdough flatbread.

## 2. Materials and Methods

### 2.1. Chemical and Reagents

The following chemicals and reagents were used in this study: β-phenylethylamine hydrochloride, tryptamine, putrescine dihydrochloride, cadaverine dihydrochloride, histamine dihydrochloride, tyramine hydrochloride, spermidine trihydrochloride, spermine tetrahydrochloride, l-lysine monohydrochloride, l-tyrosine, l-ornithine hydrochloride, ±catechin hydrate, vanillin, ammonium acetate, ammonium hydroxide solution, glycerol, Folin–Ciocalteu’s phenol reagent (≥98%), and perchloric acid (70%) (all purchased from Sigma-Aldrich Chemical Co., St. Louis, MO, USA); 2,2-diphenyl-1-picrylhydrazyl (DPPH) (Alfa Aesar, Haverhill, MA, USA); acetonitrile HPLC grade (Honeywell, SK Chemicals, Ulsan, Republic of Korea); and acetone and l-histidine hydrochloride (Samchun Pure Chemical Co., Ltd., Pyeongtaek, Republic of Korea). All chemicals and reagents used in this study were of analytical grade.

### 2.2. Bacterial Strains and Culture Preparation

Type strains of *Lactiplantibacillus plantarum* KCTC 3108, *L. fermentum* KCTC 3112, and *L. brevis* KCTC 3498 were obtained from the Korean Collection for Type Cultures (KCTC; Daejeon, Republic of Korea), and *Weissella cibaria* KCCM 41287 was purchased from the Korean Culture Center of Microorganism (KCCM; Seoul, Republic of Korea). Active strains were obtained by inoculating at 20% (*v*/*v*) in de Man, Rogosa, and Sharpe (MRS, Laboratories Conda Co., Madrid, Spain) broth, incubated for 24 h at 37 °C, and stored as glycerol stock (final concentration of 20%) at −70 °C until further use.

To prepare the cell culture inoculant, 100 µL of each bacterial glycerol stock was subcultured twice in 5 mL of MRS broth and incubated at 37 °C for 48 h. After incubation, the activated broth was suspended with 250 mL of MRS broth and incubated under the same condition. To achieve a 9-fold cell concentration, the bacterial cells were harvested by centrifugation at 8000× *g* for 5 min at 4 °C (1736R, Labogene, Seoul, Republic of Korea). The resulting cell pellet was washed twice and resuspended using sterile M/15 Sörensen’s phosphate buffer (PBS; pH 7.00; 5.675 g of Na_2_HPO_4_, 3.630 g of KH_2_PO_4_, 1 L deionized water, all obtained from Sigma-Aldrich). Microbiological enumerations were determined on MRS agar, and the final concentration was adjusted to 9 log CFU/mL.

### 2.3. Preparation of Kisra Bread

**Figure 1:**
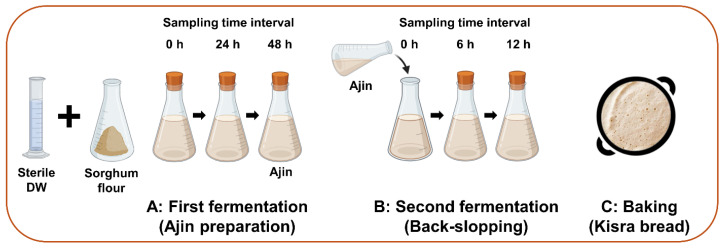
Schematic illustration of kisra processing. (**A**) A 100 g of sorghum flour was mixed with 200 mL of sterilized deionized water and fermented at 37 °C for 48 h (Section 2.3.1). (**B**) A 100 g of sorghum flour was mixed with 200 mL of sterilized deionized water and inoculated with 30 g of sourdough “ajin”, and then fermented at 37 °C for 12 h (Section 2.3.2). (**C**) Around 200 g of the back-slopping dough was poured into preheated iron plate (150 °C) and baked for 60 s (Section 2.3.2).

Fermented kisra bread was prepared according to the traditional method practiced in the Sudanese household as described by Mohammed et al. [7] with minor modification (Figure 1). The pH, water activity, total titratable acidity, and microbial count of the samples were determined immediately, whereas the remaining samples were frozen at −20 °C for further experiments. The kisra samples were divided into the following five groups: control group (kisra naturally fermented without an inoculum), LP group (kisra fermented with *L. plantarum*), LF group (kisra fermented with *L. fermentum*), LB group (kisra fermented with *L. brevis*), and WC group (kisra fermented with *W. cibaria*). The LAB count of each of the inoculated group was adjusted to 7 log CFU/g. The sorghum flour used in the inoculated groups was sterilized at 100 °C for 45 min.

#### 2.3.1. First Fermentation (Sourdough—Ajin)

Briefly, under sterile conditions, starter sourdough “ajin” was prepared traditionally by mixing 100 g of sorghum flour (Bob’s Red Mill, Milwaukee, OR, USA) with 200 mL of sterile distilled water (ratio of 1:2 (*w*/*v*)) in a covered conical flask and fermented at 37 °C for 48 h. The samples were taken aseptically at 0, 24, and 48 h.

#### 2.3.2. Second Fermentation (Back-Slopping)

The back-slopping technique was used in which 30 g of sourdough was mixed with 100 g of sorghum flour and 200 mL of water as the first fermentation. The sample was fermented at 37 °C for 12 h. The samples were taken aseptically at 0, 6, and 12 h. At the end of the second fermentation, about 200 g of fermented batter was spread on a hot iron plate (150 °C) and baked for 60 s.

### 2.4. Physicochemical and Microbiological Measurements

#### 2.4.1. pH and Water Activity

Briefly, 2 g of each sample was suspended in 18 mL of distilled water and homogenized using a vortex (Vortex-Genie, Scientific Industries, Bohemia, NY, USA). The pH of the samples was determined using a precalibrated Orion 3-star Benchtop pH meter (Thermo Scientific, Waltham, MA. USA). The water activity (a_w_) was determined using a water activity meter (AquaLab Pre; Meter Group, Inc., Pullman, WA, USA).

#### 2.4.2. Total Titratable Acidity

The total titratable acidity as lactic acid was determined using the standard titration method according to the AOAC method [22]. A 10 mL diluted sample (triplicate) was titrated against 0.1 N NaOH to the endpoint. Each 1 mL of 1 N NaOH is equivalent to 90.08 mg of lactic acid.

#### 2.4.3. Enumeration of LAB

LAB counts were determined by surface plating on MRS agar, and 100 µL samples of suitable dilutions prepared in sterile buffered peptone water (0.1% peptone (*w*/*v*), 0.85% NaCl (*w*/*v*)) were spread onto MRS agar plates and incubated aerobically for 48 h at 37 °C. The LAB count was recorded as an average and expressed as log colony-forming units per gram (log CFU/g).

### 2.5. Quantification of BAs

#### 2.5.1. Preparation of Standard Amine Solution

Standard stock solutions of β-phenylethylamine, tyramine, putrescine, cadaverine, histamine, spermidine, and spermine were separately prepared at 10,000 ppm concentration in milli-Q water. Tryptamine was prepared by dissolving in 5 mL of perchloric acid (0.4 M) according to manufacturer’s instructions. Concentrations of 10, 50, 500, and 1000 ppm were prepared from the BA standard stock solutions and used as standard peaks in HPLC analysis. An internal standard of 1,7-diaminoheptane (1 mg/mL; Sigma) under the same concentration was prepared and separated under the same chromatographic condition. Limits of detection and quantification for all BAs were 0.10 µg/mL in standard solutions. In food matrices, detection limits ranged from 0.01 to 0.10 mg/kg and quantification limits from 0.02 to 0.31 mg/kg [23].

#### 2.5.2. Derivatization of Standard BA Solutions and Sample Extracts

Extraction and determination of BAs in fermented kisra samples, kisra breads, and bacterial cultures were performed according to procedures developed by Ben-Gigirey et al. [24,25] with modifications. For derivatization reaction, 1 mL of each dilution of standard BA solutions or sample extracts was mixed with 200 µL of 2 M sodium hydroxide, 300 µL of saturated sodium bicarbonate, and 2 mL of dansyl chloride prepared in acetone. The reaction mixture was then incubated for 45 min at 40 °C. The derivatization reaction was stopped by adding 100 µL of 25% ammonium hydroxide for removing dansyl chloride residual, after which the mixture was reacted at 25 °C for 30 min in the dark. The final volume of the mixture was adjusted to 5 mL with acetonitrile. Finally, the mixture was centrifuged at 3000× *g* for 5 min, and the supernatant was filtered through 0.20-µm-pore size hydrophobic syringe filters (Millipore Co., Bedford, MA, USA).

#### 2.5.3. Chromatographic Separation of BAs by RP-HPLC

Chromatographic analysis was performed by HPLC (YL9100, YL Instruments Co., Ltd., Anyang, Republic of Korea) equipped with a UV/Vis detector (YL Instruments) and Autochro-3000 data system (YL Instruments). The RP-HPLC system was applied with a Nova-Pak C_18_ column with dimensions of 150 mm × 3.9 mm × 4 µm and kept at 40 °C. The mobile phases were 0.1 M ammonium acetate filtered through a 0.45 µm pore size PVDF membrane (Sigma) and degassed before use (eluant A) and HPLC-grade acetonitrile (eluant B) set at a flow rate of 1 mL/min. The linear gradient elution was set as follows: 0 min 50% eluant B, 19 min 90% eluant B, 20 min 50% eluant B, and 25 min 50% eluant B. The total chromatographic run was completed in 25 min with an injection volume of 10 µL and monitored at 254 nm. BAs were identified by comparing the retention times of the peaks in the samples with those of the standard solutions.

### 2.6. Antioxidant Compound Assays

#### 2.6.1. Preparation of Extracts

Methanolic extracts of the samples were obtained by adding 18 mL of methanol (SK Chemicals) to each sample at ambient temperature and extracted for 24 h in the dark. After extraction, the sample mixture was centrifuged at 6535× *g* for 15 min at 4 °C and filtered through a Whatman paper No. 1 (Whatman International Ltd., Maidson, UK). The extract samples were stored at −20 °C and used within 72 h.

#### 2.6.2. DPPH Free Radical Scavenging Activity

Antioxidant activity was evaluated based on the radical scavenging effect of stable DPPH free radical activity using the modified method of Braca et al. [26]. Briefly, a solution of 0.004% DPPH was prepared in methanol, and 1 mL of the solution was mixed with 1 mL of sample methanolic extract. The mixtures were placed in the dark for 30 min, and the optical density was measured at 517 nm using a spectrophotometer (Lambda 35; PerkinElmer Ltd., Waltham, MA, USA). Then, the percentage of inhibition of DPPH scavenging activity was calculated.

#### 2.6.3. Total Phenolic Content

The total phenolic content was determined using Folin–Ciocalteu’s modified method as described by Singleton and Rossi [27]. Briefly, a series of gallic acid dilutions (Samchun) were prepared to generate the standard calibration curve. Sample extracts and gallic acid dilutions were mixed with 750 µL of Folin–Ciocalteu’s phenol reagent (50% [*v*/*v*] dilution with deionized water) separately and reacted for 3 min in the dark. Next, 600 µL of 2% sodium carbonate was added, and the mixture was incubated in the dark for 30 min at room temperature. Absorbance was measured at 750 nm using a 96-well microplate spectrophotometer. The results of the total phenolic content were expressed in µg of gallic acid equivalent per gram of wet samples (GAE/g).

#### 2.6.4. Tannin Content

Tannin content was analyzed using the modified HCl–vanillin assay as described by Hong et al. [28]. Dilutions of catechin standard solution were prepared to generate the calibration curve. The sample extracts and working reagent were placed in a water bath at 30 °C for 10 min before use. Next, 20 µL each of sample extract and catechin stock standard dilutions was added to 30 µL of methanol and 150 µL of vanillin working reagent separately. The vanillin working reagent was freshly prepared by mixing equal volumes of 1% (*w*/*v*) vanillin solution and 8% (*v*/*v*) HCl in methanol. The tannin content was expressed as µg of catechin equivalents per gram of wet samples.

### 2.7. Isolation and Characterization of BA Producers from Naturally Fermented Kisra

#### 2.7.1. Screening for BA Producers

The amino acid decarboxylation capability was screened using decarboxylase medium developed and reported by Bover-Cid and Holzapfel [12]. Briefly, 5 g of 24 h fermented kisra sample was mixed with 45 mL of sterile buffered peptone water. The mixture was then homogenized and serially diluted, after which 100 µL suitable dilutions were plated onto the decarboxylase medium supplemented with 0.5% l-tyrosine, 0.005% pyridoxal-5-phosphate, and 0.006% bromocresol purple (Sigma) as a color indicator (pH 5.30). The plates were incubated at 37 °C both aerobically and anaerobically using an atmospheric combination of 95% nitrogen and 5% hydrogen (anaerobic chamber, Coy Lab. Products, Inc., Grass Lake, MI, USA). After incubation, three different morphologies were observed on the cultured plates, and a random selection of each morphology was subcultured by streaking onto new decarboxylase medium and incubating under the same conditions. The decarboxylation activities of isolated strains were demonstrated by the color change to purple, indicating alkalic compound production (i.e., BAs).

#### 2.7.2. Isolation and Characterization of BA Producers

Grouped based on morphological differences (see Section 2.7.1), a total of 123 Group A isolates tested positive for tyramine production (intense color change), 50 Group B isolates tested negative (no color change), and 40 group C isolates tested positive (intense color change). Isolates of Groups A and B, presumably LAB strains, were isolated from the decarboxylase medium and streaked onto MRS agar. After incubation for 48 h at 37 °C, individual colonies were purified by streaking onto fresh MRS plates and incubated under the same conditions. Characterization of Group A and B isolates was performed using microscopic morphology, m-Enterococcus selective agar (m-EA; MB Cell, Seoul, Republic of Korea), and pyrrolidonyl arylamidase (PYR) test kits (Hardy Diagnostics, Santa Maria, CA, USA). Group A isolates were able to grow on m-EA and tested positive (pink color) on the PYR test. Accordingly, they were characterized as presumably *Enterococcus*. In contrast, Group B isolates exhibited poor growth on m-EA and tested negative (no color) on the PYR test. Therefore, they were characterized as presumably *Pediococcus*. The species-level identification of two strains belonging to Group A with the strongest tyramine production was performed through 16S rRNA gene sequence analysis, amplified with the universal bacterial primer pair, 518F (5′-CCAGCAGCCGCGGTAATACG-3′) and 805R (5′-CCCCCAGCCTAGCTTAGTTT-3′) (SolGent, Daejeon, Republic of Korea). The two strains were identified as *E. faecalis*. All LAB isolates were maintained at −70 °C as a glycerol stock. Similarly, 40 presumptive yeasts (yeast-like strains) were cultured and purified on yeast peptone dextrose (YPD) agar (1% yeast extract, 2% peptone, 2% dextrose, and 2% agar) supplemented with chloramphenicol (100 µg/L) and incubated at 30 °C for 72 h. Group C isolates were able to grow on YPD supplemented with chloramphenicol. Thus, they were characterized as presumptive yeasts. Yeast colonies were purified by subculturing on YPD agar, and after purification, the isolates were preserved at −70 °C as a glycerol stock.

#### 2.7.3. In Vitro Determination of BA Production Capability of the Isolates

A random selection for an approximate quarter of each isolate group was evaluated for its members’ BA-producing abilities as described by Ben-Gigirey et al. [24,25] with modifications. Glycerol stock activation (twice) was accomplished by inoculating 100 μL in 5 mL of MRS broth for Groups A and B and 5 mL of YPD broth for Group C. The incubation conditions were as follows: Groups A and B: 48 h at 37 °C, Group C: 72 h at 30 °C. After two rounds of enrichment, each of the cultured broths (MRS or YPD) was inoculated twice in succession at 0.1% (*v*/*v*) into the decarboxylation broth supplemented with 0.005% (*w*/*v*) pyridoxal-5-phosphate and each of the following precursor amino acids at 0.5% (*w*/*v*): l-ornithine hydrochloride, l-tyrosine, l-histidine hydrochloride monohydrate, and l-lysine monohydrochloride. The final pH of the broths was adjusted to 5.80. The abovementioned incubation conditions were applied. The extraction and quantification of BAs in the decarboxylation broth were performed as mentioned earlier in Section 2.5.

### 2.8. Statistical Analysis

All measurements were conducted in triplicate, and fermentation experiments were performed in duplicate. Results were expressed as mean ± standard deviation. Statistical analyses were conducted by one-way analysis of variance (ANOVA) using the Minitab statistical software, version 17 (Minitab Inc., State College, PA, USA). Fisher’s pairwise comparison test was used for mean comparisons at 5% probability (*p*), and *p* ˂ 0.05 was considered statistically significant.

## 3. Results and Discussion

### 3.1. Changes in Fermentation Parameters and Production of Antioxidant Compounds and BAs in Naturally Fermented Kisra

#### 3.1.1. Changes in pH, Total Titratable Acidity, and LAB Counts during Natural Fermentation of Kisra

**Figure 2:**
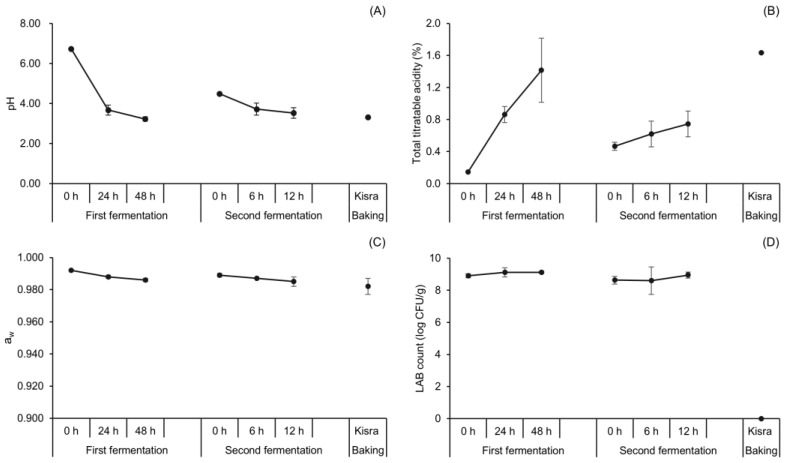
Changes in physicochemical and microbiological properties of kisra bread during natural fermentation. (**A**) pH, (**B**) total titratable acidity, (**C**) water activity, (**D**) LAB count. Error bars indicate standard deviations calculated from three trials.

The physicochemical and microbial properties of the samples were determined to ensure the success of fermentation (Figure 2). The natural fermentation group (also serving as control group in Section 3.3) is described here to separately provide baseline information on BA content in naturally fermented kisra. In the first fermentation, the pH decreased during natural fermentation from 6.74 at 0 h to 3.68 at 24 h and further decreased to 3.23 at 48 h. Conversely, the total titratable acidity increased from an initial 0.15% to 1.42% at the end of the first fermentation. This coincided with an increase in the LAB count throughout the first fermentation period from an initial 8.90 to 9.12 log CFU/g. Similarly, the back-slopping (second fermentation) revealed a comparable decrease in pH values, ranging from 4.49 to 3.53. This was paralleled by an increase in the total titratable acidity reaching 0.75% at 12 h, whereas the LAB count considerably increased from 8.63 to 8.95 log CFU/g.

The observed negative correlation between pH and total titratable acidity values during the first and second fermentation stages, respectively, is similar to that of lactic acid-fermented sorghum as described in previous studies [8,29]. Because LAB produce organic acids, it is natural that the increase in the number of LAB resulted in a decrease in pH and an increase in acidity during the fermentation of sorghum and kisra in the previous and present studies. Baking reduced the pH to 3.31 and resulted in the highest total titratable acidity of 1.64%. No LAB were detected in the baked kisra. Meanwhile, the a_w_ during the first and second fermentation ranged from 0.992 to 0.985, falling within the limits suitable for microbial growth. Baking further reduced the a_w_ levels, reaching 0.982 for kisra bread. Moreover, baking generally resulted in a decrease in the pH and a_w_ of kisra as well as an increase in the total titratable acidity compared with the corresponding 12 h second fermentation samples. This might be due to the moisture content resulting from the dry matter in kisra bread.

#### 3.1.2. Changes in Antioxidant Activity, Total Phenolic Content, and Tannin Content during Natural Fermentation of Kisra

The antioxidant activity of kisra bread was evaluated using the DPPH scavenging activity assay. Table 1 and Table S1 show a summary of the changes in the antioxidant activity, total phenolic content, and tannin content. The first fermentation caused a remarkable decrease in the DPPH scavenging activity, reaching 55.91% at 48 h from 71.04% at 0 h. The total phenolic content increased slightly from 1171.80 μg/g at 0 h to 1207.10 μg/g at 24 h in the first fermentation and then decreased to 1162.90 μg/g at 48 h. Meanwhile, the tannin content decreased significantly from 22.63 µg/g at 0 h to 17.37 µg/g at 48 h. The second fermentation resulted in a further decrease in the DPPH scavenging activity, reaching 54.61% at 12 h from 56.92% at 0 h. Similar to that in the first fermentation, the total phenolic content exhibited a pattern of fluctuation, eventually decreasing to 1118.80 μg/g at 12 h. However, the second fermentation resulted in a noticeable increase in tannin content, reaching 27.89 μg/g at 12 h. Baking substantially increased the DPPH scavenging activity to 85.16%, along with an increase in the total phenolic content to 1386.50 μg/g and tannin content to 33.16 μg/g.

The observed decrease in the DPPH scavenging activity during both the first and second fermentations is consistent with findings reported by Dlamini et al. [30], who observed a reduction in the antioxidant activity of fermented sorghum slurry and porridge. This reduction in the DPPH scavenging activity may be attributed to the decrease in the total phenolic content. A similar decrease in the total phenolic content was observed by Mehdizadeh et al. [31] during the fermentation of rambutan seeds. This may be due to the action of the polyphenol oxidase enzyme that performs oxidation reaction, diffusing the phenolics and oxidizing them [32]. Furthermore, the reduction in tannin content during the first fermentation may be attributed to its binding to protein and the resulting decrease in the extractable amount. Losses of tannin are also caused by the hydrolysis of polyphenolic compounds during fermentation. The significant increase observed in the DPPH scavenging activity, total phenolic content, and tannin content after baking is consistent with that reported by Zaroug et al. [21], who also observed an increase in the antioxidant activity and total phenolic and tannin contents in fermented baked kisra. In addition, Hithamani and Srinivasan [33] showed that roasting sorghum at high temperatures (150 °C) resulted in an increase in the average antioxidant activity and total phenolic content, which supports the observations of the present study well.

#### 3.1.3. Changes in BA Content during Natural Fermentation of Kisra

**Figure 3:**
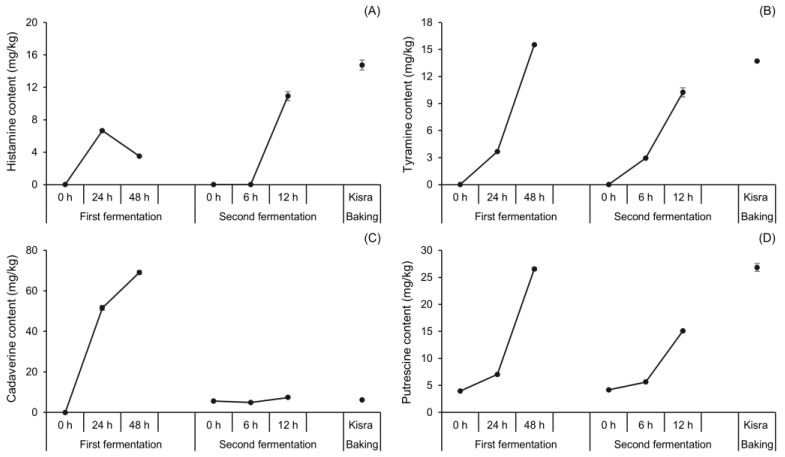
Changes in BA concentrations of kisra bread during natural fermentation. (**A**) histamine, (**B**) tyramine, (**C**) cadaverine, (**D**) putrescine. The natural fermentation group shown in this figure also served as the control group in Figure 5. Error bars indicate standard deviations calculated from three trials.

The BA-associated risks of kisra bread were evaluated by analyzing the changes in BA content during the natural fermentation of kisra bread, as shown in Figure 3. Cadaverine, putrescine, tyramine, and histamine were the primary BAs produced during the fermentation, whereas β-phenylethylamine and tryptamine were not detected, and spermidine and spermine demonstrated insignificant changes in their content at all processing stages. Therefore, the four primary BAs were mainly described hereafter. Apart from this, putrescine, spermidine, and spermine were detected at 0 h of the first fermentation, suggesting that these three amines originated from sorghum flour. Similarly, Paiva et al. [34] reported detectable amounts of putrescine, spermidine, and spermine in sorghum seeds before germination. This is generally because of the ubiquitous presence of the diamine putrescine and the polyamines spermidine and spermine in all plant cells [35].

Tyramine and histamine are BAs of interest due to their high toxicity and cause the most frequent foodborne diseases associated with BAs [36]. Figure 3A summarizes the changes in histamine content during natural fermentation. The content reached 6.64 mg/kg from an initial nondetectable (ND) level during the first 24 h of the first fermentation. It then rapidly decreased to approximately half (3.50 mg/kg) by the end of the first fermentation. These results are explained by findings from previous studies. During fermentation, histidine is converted into histamine by histidine decarboxylase enzymes, which consume protons (H^+^) and initially raise the pH [37]. Once the pH is raised, a pH-dependent histamine/histidine exchange reaction occurs in LAB cells [38], eventually reducing histamine content. A histamine content of 10.92 mg/kg was also detected at 12 h of the second fermentation. The highest histamine concentration detected in this study was 14.76 mg/kg in kisra bread. Meanwhile, the tyramine content (Figure 3B) increased rapidly during the first fermentation, reaching 3.67 and 15.52 mg/kg at 24 and 48 h, respectively. Similarly, the second fermentation led to a progressive increase in tyramine content from ND levels at 0 h to 10.26 mg/kg at 12 h. Baking increased the tyramine and histamine contents, reaching 13.73 and 14.76 mg/kg, respectively. Similarly, in the roasting process of cocoa beans, it was found that higher temperatures resulted in the highest increase in BAs due to the thermal decarboxylation of amino acids during heat treatment [39]. Another previous study [40] on soybean paste also reported that roasting was identified as a factor contributing to the elevation of BA content.

Unlike tyramine and histamine, both cadaverine and putrescine are considered to exhibit less toxicity; however, the major issue related to these amines is the potentiation of the toxicity of other amines, especially histamine. Figure 3C shows the changes in cadaverine concentration during the natural fermentation of kisra bread. The cadaverine content absolutely increased from ND levels at 0 h to 51.58 mg/kg at 24 h and further to 69.04 mg/kg at 48 h of the first fermentation. Interestingly, the second fermentation (back-slopping) resulted in a significantly (*p* ˂ 0.05) lower cadaverine content than the first fermentation (sourdough—ajin) at each sampling time. The cadaverine content slightly increased from 5.60 mg/kg at 0 h to 7.38 mg/kg at 12 h. This discrepancy may be attributed to the relatively shorter duration of the second fermentation than that of the first fermentation. Among the BAs detected in kisra bread, putrescine (Figure 3D) showed the highest concentration, reaching 26.88 mg/kg, which significantly differed that of from other amines present. Initially, the putrescine content was 3.97 mg/kg and steadily increased to 26.53 mg/kg at 48 h of the first fermentation. Similarly, the second fermentation resulted in a remarkable increase in putrescine content, peaking at 15.08 mg/kg at 12 h of the second fermentation. Although the BA levels detected, such as histamine and tyramine, in naturally fermented kisra were less than the risk limit, BA-associated risks may arise because of the high daily intake of kisra and the use of aged sourdough that may have a higher BA content. These risks could include headaches, nausea, vomiting, diarrhea, hypertension, and allergic reactions [13] However, no such adverse events have been yet reported in the local community. Therefore, it is crucial to determine the causative agents of BA formation in kisra and determine methods for reducing their levels.

### 3.2. Determination of the Causative Agents of BA Formation in Kisra

As clarified in Section 3.1.3, the spontaneous fermentation of kisra resulted in a remarkable increase in tyramine content. Therefore, the causative agents of BA (particularly tyramine) formation in kisra were determined. For this purpose, a total of 213 strains isolated from 24 h fermented kisra dough were separated into three groups—A, B, and C (123, 50, and 40 isolates, respectively)—according to morphological characteristics. Based on the positive results obtained from m-EA selective medium and PYR test, Group A was tentatively the *Enterococcus* genus. In contrast, the negative PYR test results for group B, combined with the m-EA medium manufacturer’s information, a remarkable “possibility of *Pediococcus* growth”, supported the possibility of this group being the *Pediococcus* genus. This finding showed that the *Enterococcus* genus was dominant during the 24 h fermentation, as it accounted for the majority of the bacterial isolates, although the *Pediococcus* genus was also present, which is partially consistent with the results of a previous study [8] in which *Pediococcus* was more prevalent than *Enterococcus* during the 24 h fermentation of kisra. In the meantime, isolates belonging to Group C isolated using YPD medium supplemented with chloramphenicol were tentatively characterized as yeasts. Subsequently, the possible contribution of isolate groups to BA production during kisra fermentation was assessed through in vitro BA production tests combined with a preliminary screening using a decarboxylase medium, as shown in Table 2. The preliminary screening for tyramine producers yielded a positive (strong color change) result for all isolates in group A. The in vitro BA production tests for the group showed a high tyramine production with an average of 184.19 µg/mL, while other BAs were at low concentrations that could be considered insignificant. It is noteworthy that the two strains with the strongest tyramine productivity were identified as *E. faecalis* through 16S rRNA gene sequencing. Conversely, Group B isolates exhibited a negative (no color change) result in the preliminary screening. Compatibly, they demonstrated substantially weak in vitro BA production, averaging at 0.07 µg/mL for histamine, 0.91 µg/mL for tyramine, 0.08 µg/mL for cadaverine, and 0.39 µg/mL for putrescine. Group C isolates exhibited comparatively weak in vitro tyramine production with an average of 1.41 µg/mL, despite positive results in the preliminary screening. The in vitro analysis also revealed a weak histamine production of 0.22 µg/mL for these isolates.

For Group C, exceptional difference in tyramine productivity between preliminary screening and quantitative analysis has also been observed in previous studies [41,42,43] that have reported false-positive reactions due to the production of other alkaline compounds in the medium. In the present study, the perceived false positive results might be attributed to the production of putrescine and cadaverine, which could be confirmed through in vitro analysis. Meanwhile, more than half of Group C isolates demonstrated high putrescine production with an average of 355.60 µg/mL, whereas the remaining showed considerably low production of ≤4 µg/mL. Interestingly, the Group C isolates that exhibited high putrescine production also demonstrated considerable cadaverine production, with an average of 8.14 µg/mL, and vice versa. Therefore, among the three isolate groups, Group A accounted for the highest tyramine production and the highest ratio of isolates which implies that *Enterococcus* genus might be responsible for tyramine accumulation during kisra fermentation. Conversely, Group B isolates with weak BA production seemed to have potential as starter cultures for BA reduction during kisra fermentation. In addition, Group C exhibited the highest production capability of both putrescine and cadaverine, which indicates that yeasts may significantly contribute to the accumulation of these two diamines.

### 3.3. Effect of LAB Species on Fermentation Parameters and Production of Antioxidant Compounds and BAs during Kisra Fermentation

To inhibit BA accumulation during kisra fermentation, the potential of LAB species commonly used as commercial starters was investigated. For this purpose, four representative LAB species that have been confirmed to be dominant in sorghum fermentation were selected [44,45,46]. Moreover, when applying the LAB species for kisra fermentation, along with their BA-reducing effect, the changes in fermentation parameters and antioxidant compound production were observed in the groups inoculated with LAB species (LAB groups, including LP, LF, LB, and WC groups).

#### 3.3.1. Effect of LAB Species on pH, Total Titratable Acidity, and LAB Counts during Kisra Fermentation

**Figure 4:**
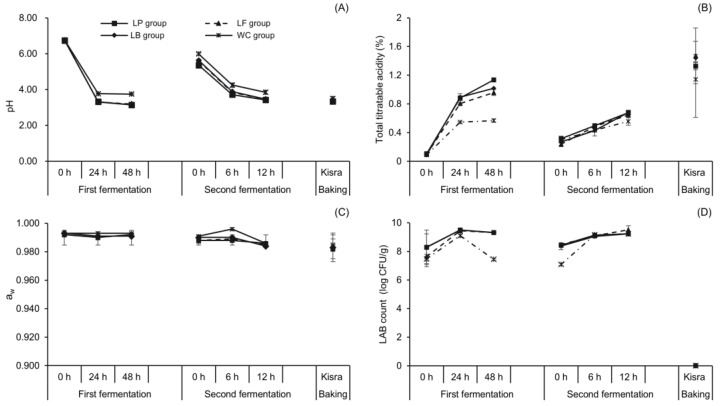
Changes in physicochemical and microbiological properties during kisra fermentation with different lactic acid bacterial species. (**A**) pH, (**B**) total titratable acidity, (**C**) water activity, (**D**) LAB count. ■: LP group (kisra fermented with *L. plantarum*), ▲: LF group (kisra fermented with *L. fermentum*), ♦: LB group (kisra fermented with *L. brevis*), Ж: WC group (kisra fermented with *W. cibaria*). Error bars indicate standard deviations calculated from three trials.

To investigate the fermentation dynamics of kisra with the application of LAB species, the physiochemical and microbial changes during kisra fermentation were observed, as shown in Figure 4 and Table S2. In the first fermentation, the initial pH values ranged from 6.74 to 6.77 in all groups inoculated with different LAB species. Subsequently, at 48 h, the pH values in the LP, LF, LB, and WC groups decreased to 3.14, 3.19, 3.15, and 3.75, respectively. The pH value in the WC group was significantly (*p* ˂ 0.05) higher than that in the other groups at 48 h of the first fermentation, which was associated with a lower LAB count of 7.44 log CFU/g compared to 9.31 log CFU/g in the LP group, 9.32 log CFU/g in the LF group, and 9.31 log CFU/g in the LB group. The total titratable acidity increased until the end of the first fermentation, reaching 1.13%, 0.96%, 1.02%, and 0.57% in the LP, LF, LB, and WC groups, respectively. Similar to that in the first fermentation, the second fermentation resulted in a similar decrease in pH from 3.42 to 3.85 at 12 h, and the WC group showed the highest pH among the groups. The LAB counts in the LP, LB, and WC groups were statistically comparable, with an average of 9.23 log CFU/g at 12 h of the second fermentation, whereas the LF group showed the highest LAB count of 9.52 log CFU/g. Unlike in the first fermentation, in the second fermentation, no clear correlation was observed between pH and LAB counts, which might be because of the relatively short fermentation period. The total titratable acidity increased until the end of the second fermentation, with no significant difference observed among the LP (0.68%), LF (0.64%), and LB (0.68%) groups. Nevertheless, the WC group showed a considerably low total titratable acidity of 0.56%.

The significantly higher pH and low LAB counts observed in the WC group at 48 h of the first fermentation suggested that *W. cibaria* did not thrive under acidic conditions or that the growth of *W. cibaria* was slower than that of other LAB species. In support of the first assumption, Ricciardi et al. [47] reported that the kinetic growth of *W. cibaria* ceased after 48 h of fermentation at pH < 4.00. Furthermore, the reduced total titratable acidity observed in the WC group at the end of both fermentation stages might be attributed to the heterofermentative nature and low lactic acid productivity of the strain. Jang et al. [48] and Maślak et al. [49] also reported lower production of lactic acid by *W. cibaria* than by other heterofermentative LAB species. Considering such changes in fermentation parameters, the *Lactobacillus* species tested in this study is more preferable for kisra fermentation. Baking, like natural fermentation, reduced the pH of the LP, LF, and LB groups to 3.34 and to 3.50 in the WC group. The total titratable acidity increased to 1.33%, 1.47%, 1.44%, and 1.14% in the LP, LF, LB, and WC groups, respectively. No LAB were detected in all groups, which was because of the high baking temperature. The a_w_ of all LAB groups ranged from 0.993 to 0.982 during kisra processing.

The LP, LF, and LB groups demonstrated high LAB counts and low pH values at the end of both fermentation stages compared with values observed in the control group. These results highlight the efficacy of the LAB species used in this study, particularly *L. plantarum*, *L. fermentum*, and *L. brevis*, in improving the fermentation dynamics of kisra.

#### 3.3.2. Effect of LAB Species on Antioxidant Activity, Total Phenolic Content, and Tannin Content during Kisra Fermentation

The functionality of the LAB groups was evaluated by investigating the DPPH scavenging activity, total phenolic content, and tannin content (Table 3). During the first and second fermentation, the DPPH scavenging activity increased in all LAB groups, whereas it decreased in the control group. The tannin content in all LAB groups fluctuated throughout both fermentation stages, with variations observed among different LAB groups. In particular, both DPPH scavenging activity and tannin content increased during baking across all LAB groups, although no significant (*p* > 0.05) difference was observed compared to the control group. Overall, the LAB species exerted no significant impact on the DPPH scavenging activity and tannin content of kisra bread. Conversely, the total phenolic content of kisra bread significantly (*p* ˂ 0.05) increased after using LAB species compared with that in the control group. In this case, the first fermentation resulted in an increase in the total phenolic content, reaching 1257.10, 1292.40, 1239.40, and 1357.10 µg/g in the LP, LF, LB, and WC groups at 48 h, respectively. During the second fermentation, the total phenolic content increased in the LP and WC groups to 1286.50 and 1280.60 µg/g, respectively, at 12 h, whereas it decreased slightly in the LF and LB groups to 1218.80 and 1118.80 µg/g, respectively. Baking significantly (*p* ˂ 0.05) increased the total phenolic content up to 20% compared to the control group (1386.50 µg/g). The content was 1668.80 µg/g in the WC group, 1610.00 µg/g in the LP group, and 1574.7 µg/g in the LF group. After baking, the LB group exhibited a relatively lower phenolic content of 1433.50 µg/g than that of other LAB groups.

The increase in the DPPH scavenging activity observed during the fermentation of LAB groups was probably due to the accumulation of various antioxidant compounds and metabolites, particularly free soluble phenolic molecules. This assumption is supported by the direct relationship between the increase in DPPH scavenging activity and total phenolic content during fermentation in both previous study [50] and the present study. The increase in the total phenolic content may be attributed to the enhanced bioavailability of phenolic compounds induced by cell wall-degrading enzymes of fermentation microbes [51,52]. Furthermore, unlike the increase in the total phenolic content in the LP and WC groups, the slight decrease in the total phenolic content in the LF and LB groups during the second fermentation may be attributed to the microbial degradation or oxidation of diffused phenolic compounds [53]. Further research is necessary to confirm the differences in degradation activity depending on the LAB species. Meanwhile, baking generally increased the DPPH scavenging activity, total phenolic content, and tannin content. These results are supported by the fact that high temperatures during baking can cause the breakdown of high-molecular-weight polyphenols, such as condensed tannins, and convert them into low-molecular-weight forms that are more extractable [54]. Randhir et al. [55] also reported that roasting causes the release of bound phenolic compounds.

#### 3.3.3. Effect of LAB Species on BA Content during Kisra Fermentation

**Figure 5:**
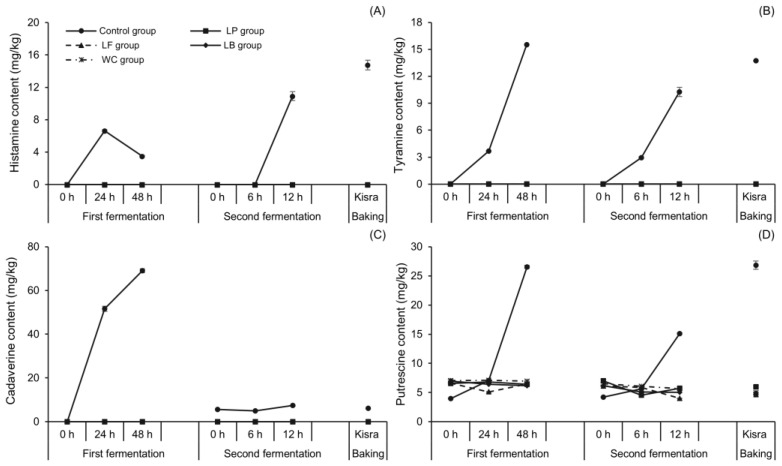
Reduction in BA concentrations during kisra fermentation with different lactic acid bacterial species. (**A**) histamine, (**B**) tyramine, (**C**) cadaverine, (**D**) putrescine. The control group shown in this figure also served as the natural fermentation group in Figure 3. ●: control group (kisra naturally fermented without an inoculum), ■: LP group (kisra fermented with *L. plantarum*), ▲: LF group (kisra fermented with *L. fermentum*), ♦: LB group (kisra fermented with *L. brevis*), Ж: WC group (kisra fermented with *W. cibaria*). Error bars indicate standard deviations calculated from three trials.

To investigate the BA-reducing effect of LAB species applied to kisra fermentation, the changes in BA content were observed throughout all stages, including the first and second fermentation and baking, as shown in Figure 5. In the control group for kisra bread, the content of putrescine was the highest, followed by histamine, tyramine, and cadaverine (Section 3.1.3 or Figure 5). However, the use of LAB species in kisra fermentation significantly inhibited the formation of putrescine and eliminated the formation of cadaverine, tyramine, and histamine throughout all stages. Moreover, neither β-phenylethylamine nor tryptamine was detected at any of the processing stages.

Unlike the other primary BAs, whose formation was completely suppressed throughout all stages, putrescine formation exhibited slightly different change patterns depending on the LAB species at each stage. During the first fermentation, the putrescine content in the LP group slightly increased from 6.55 mg/kg at 0 h to 6.80 mg/kg at 24 h and then decreased to 6.40 mg/kg at 48 h. In the LF group, the putrescine content decreased from 6.85 mg/kg at 0 h to 5.11 mg/kg at 24 h and then increased to 6.48 mg/kg at 48 h. In the LB group, it showed an overall decrease from 6.95 mg/kg at 0 h to 6.17 mg/kg at 48 h. In the WC group, the putrescine content remained constant with an insignificant change from 7.04 mg/kg at 0 h to 6.94 mg/kg at 48 h. During the second fermentation, the putrescine content in the LP group decreased from 6.99 mg/kg at 0 h to 4.54 mg/kg at 6 h but increased to 5.72 mg/kg at 12 h. In the LF, LB, and WC groups, it decreased from 6.10, 6.95, and 6.49 mg/kg at 0 h to 3.98, 5.03, and 5.67 mg/kg, respectively, at 12 h. The LF group showed the highest reduction rate (approximately 35%) of putrescine content. After baking, the putrescine content was lowest in the LF group at 4.63 mg/kg, followed by 4.87 mg/kg in the LB group, 5.00 mg/kg in the WC group, and 5.99 mg/kg in the LP group. Consequently, the use of LAB species significantly reduced the content to less than about 23% compared to naturally fermented kisra.

Meanwhile, the ability of LAB groups to inhibit the formation of cadaverine, tyramine, and histamine is consistent with previous studies. For instance, Bartkiene et al. [56] observed a similar reduction in cadaverine levels during the fermentation of lupine flour with *P. acidilactici*. In their study, cadaverine was not detected in the sourdough fermented with *P. acidilactici*, whereas its content in spontaneously fermented sourdough was 54.7 mg/kg. Similarly, Polak et al. [57] reported that a commercial starter culture containing LAB species decreased the formation of tyramine in chickpea sourdough to <0.1 mg/100 g. Furthermore, Bartkiene et al. [58] showed that *Lactobacillus-sakei*-fermented soybean had a significantly lower histamine content (10.9 mg/kg) than spontaneously fermented soybean (41.5 mg/kg). Therefore, the use of appropriate LAB species/strains may be a promising approach to improve the safety of kisra bread associated with the formation of BAs.

The minimal variations observed in the content of polyamines (spermidine and spermine) in both the control and LAB groups across the first and second fermentation stages (Table S3) may not be attributed to the fermentation process. Instead, such variations may be caused by changes in water content during fermentation, as most polyamines are water-soluble, which significantly impacts their solubility and distribution within the sample [59]. Moreover, baking resulted in an increase in spermidine and spermine content, which may also be attributed to the lower water content of kisra bread. The spermidine content in kisra bread was also significantly lower (*p* ˂ 0.05) in all LAB groups than in the control group, whereas the content of putrescine and spermine was remarkably higher in all LAB groups. Such differences in polyamine content between the control group and LAB groups may be due to the sterilization of sorghum flour for the inoculation of LAB species rather than the fermentation process. In other words, during heat treatment, the spermidine present in sorghum flour was decomposed to produce putrescine and its intermediates, and then the decomposition products were condensed to form spermine. Further studies are required to clarify the action of heat treatments on polyamines.

## 4. Conclusions

BAs were detected in naturally fermented kisra bread, with cadaverine being the most prevalent in the fermented “ajin” sourdough, whereas putrescine was the most abundant in both back-slopping dough and kisra bread. Histamine level was the highest in kisra bread, and tyramine level was the highest in the fermented “ajin” sourdough. However, the histamine and tyramine levels detected in fermented kisra samples and kisra bread were less than the safety limits suggested by Brink et al. [60] for histamine and tyramine concentration in food. In vitro BA production tests on microorganisms isolated from naturally fermented kisra dough revealed that the *Enterococcus* genus had a prolific ability to produce tyramine and was probably responsible for tyramine production in kisra bread. Presumptively yeast exhibited strong in vitro production of cadaverine and putrescine, so it may significantly contribute to their production in kisra bread. Interestingly, isolates of the genus *Pediococcus* had potential to be used as a starter culture, as they could produce lower amounts of BAs in the in vitro tests. Nevertheless, when using *Pediococcus* as a starter culture, it is necessary to consider its effect on qualities such as the organoleptic properties of kisra bread.

In the fermentation experiments, the LP, LF, and LB groups achieved the optimal pH and total titratable acidity required for the sensory quality of kisra bread. Unlike that in the other LAB groups, the pH and total titratable acidity of the WC group deviated significantly, rendering it unsuitable for kisra fermentation. The fermentation of LAB groups exerted no significant effect on the DPPH scavenging activity or tannin content in kisra bread compared with natural fermentation. Nevertheless, it significantly increased the total phenolic content, potentially providing additional health benefits. Furthermore, all LAB groups were capable of inhibiting and/or reducing BA formation during kisra fermentation—that is, a complete inhibition of histamine, tyramine, and cadaverine formation (100%) and a significant (*p* ˂ 0.05) reduction in putrescine content were observed. These findings suggest that the LAB species used in this study, except for *W. cibaria*, can significantly improve the safety, quality, and functionality of kisra bread by controlling BA formation and increasing the total phenolic content. Therefore, this study provides selection criteria at the species level for LAB strains that can be used in the industrial production of kisra bread.

## Figures and Tables

**Table 1:** Changes in DPPH scavenging activity, total phenolic content, and tannin content of kisra bread during natural fermentation.

Processing Stages	Antioxidant Parameters		
	DPPH (%)	TPC (μg/g) ^1^	Tannin (μg/g) ^2^
First fermentation			
0 h	71.04 ± 1.43 ^3,b^	1171.80 ± 33.30 ^bc^	22.63 ± 0.00 ^ab^
24 h	55.19 ± 2.65 ^cd^	1207.10 ± 8.30 ^b^	17.37 ± 0.00 ^b^
48 h	55.91 ± 0.82 ^cd^	1162.90 ± 29.10 ^bc^	17.37 ± 0.00 ^b^
Second fermentation			
0 h	56.92 ± 2.24 ^cd^	1198.20 ± 20.80 ^b^	17.37 ± 0.00 ^b^
6 h	59.08 ± 2.04 ^c^	1233.50 ± 29.10 ^b^	20.00 ± 3.70 ^ab^
12 h	54.61 ± 1.02 ^d^	1118.80 ± 49.90 ^c^	27.89 ± 7.40 ^ab^
Baking			
Kisra bread	85.16 ± 0.61 ^a^	1386.50 ± 37.40 ^a^	33.16 ± 14.90 ^a^

^1^ Total phenolic content is expressed as µg of gallic acid equivalent/g of wet samples. ^2^ Tannin content is expressed as µg of catechin equivalents/g of wet samples (Vanillin-HCl method). ^3^ Data represented as mean ± standard deviation from triplicate measurements. Values in the same column followed by different letters (a–d) are significantly different (*p* < 0.05). The natural fermentation group shown in this table also served as the control group in Table 3.

**Table 2:** In vitro BA production of bacteria and yeasts isolated from kisra sourdough.

Groups	N_T_ ^1^	N_A_ ^2^	Characterization of Isolate ^3^	BA-Producing Intensity	BA Productivity (µg/mL) ^4^			
					PUT	CAD	HIS	TYR
A	123	34	Tentative *Enterococcus*	Strong producer	0.22 ± 0.04 (0.13–0.38) ^5^	0.16 ± 0.05 (0.11–0.32)	0.21 ± 0.17 (0.09–0.68)	184.19 ± 13.60 (168.30–212.80)
B	50	12	Tentative *Pediococcus*	Weak producer	0.39 ± 0.03 (0.36–0.43)	0.08 ± 0.007 (0.07–0.09)	0.07 ± 0.08 (ND ^6^–0.67)	0.91 ± 0.25 (0.63–1.34)
C	40	7	Tentative Yeast	Strong producer	355.60 ± 51.30 (434.90–297.37)	8.14 ± 3.71 (3.24–13.09)	0.22 ± 0.13 (ND–0.34)	1.41 ± 0.23 (0.98–1.85)
		5		Weak producer	3.82 ± 0.47 (3.65–4.06)	2.16 ± 0.67 (1.43–2.85)	ND	

^1^ N_T_: the number of strains isolated. ^2^ N_A_: the number of strains analyzed. ^3^ The characterization of the isolates analyzed was conducted using m-EA selective medium, PYR test, and microscopic morphology. ^4^ PUT: putrescine, CAD: cadaverine, HIS: histamine, TYR: tyramine. ^5^ Data represented as mean ± standard deviation (production range from minimum to maximum). ^6^ ND: no production detected.

**Table 3:** Changes in DPPH scavenging activity, total phenolic content, and tannin content during kisra fermentation with different lactic acid bacterial species.

Groups ^1^	Processing Stages		Antioxidant Parameters		
			DPPH (%)	TPC (μg/g) ^2^	Tannin (μg/g) ^3^
Control group	First Fermentation	0 h	71.04 ± 1.43 ^4,b^	1171.80 ± 33.30 ^bc^	22.63 ± 0.00 ^ab^
		24 h	55.19 ± 2.65 ^cd^	1207.10 ± 8.30 ^b^	17.37 ± 0.00 ^b^
		48 h	55.91 ± 0.82 ^cd^	1162.90 ± 29.10 ^bc^	17.37 ± 0.00 ^b^
	Second Fermentation	0 h	56.92 ± 2.24 ^cd^	1198.20 ± 20.80 ^b^	17.37 ± 0.00 ^b^
		6 h	59.08 ± 2.04 ^c^	1233.50 ± 29.10 ^b^	20.00 ± 3.70 ^ab^
		12 h	54.61 ± 1.02 ^d^	1118.80 ± 49.90 ^c^	27.89 ± 7.40 ^ab^
	Baking	Kisra	85.16 ± 0.61 ^a^	1386.50 ± 37.40 ^a^	33.16 ± 14.90 ^a^
LP group	First Fermentation	0 h	56.77 ± 0.41 ^e^	1098.20 ± 4.20 ^d^	17.37 ± 7.40 ^ab^
		24 h	67.00 ± 0.20 ^c^	1183.50 ± 49.90 ^c^	17.37 ± 0.00 ^b^
		48 h	73.20 ± 2.04 ^b^	1257.10 ± 45.80 ^b^	17.37 ± 0.00 ^b^
	Second Fermentation	0 h	62.82 ± 0.41 ^d^	1177.60 ± 8.30 ^c^	22.63 ± 7.40 ^b^
		6 h	67.15 ± 0.41 ^c^	1174.70 ± 12.50 ^c^	14.74 ± 3.70 ^ab^
		12 h	69.45 ± 1.63 ^c^	1286.50 ± 4.20 ^b^	20.00 ± 3.70 ^ab^
	Baking	Kisra	82.28 ± 1.02 ^a^	1610.00 ± 29.10 ^a^	35.79 ± 18.60 ^a^
LF group	First Fermentation	0 h	56.77 ± 2.45 ^f^	1174.70 ± 20.80 ^d^	12.11 ± 7.40 ^b^
		24 h	68.16 ± 1.02 ^cd^	1239.40 ± 20.80 ^cd^	12.11 ± 0.00 ^b^
		48 h	73.05 ± 0.20 ^b^	1292.40 ± 20.80 ^bc^	33.16 ± 7.40 ^ab^
	Second Fermentation	0 h	63.54 ± 1.83 ^e^	1365.90 ± 8.30 ^b^	12.11 ± 7.40 ^b^
		6 h	65.71 ± 2.45 ^de^	1262.90 ± 54.10 ^c^	22.63 ± 7.40 ^ab^
		12 h	69.60 ± 1.02 ^bc^	1218.80 ± 16.60 ^cd^	27.89 ± 0.00 ^ab^
	Baking	Kisra	83.72 ± 0.61 ^a^	1574.7 ± 62.40 ^a^	38.42 ± 22.30 ^a^
LB group	First Fermentation	0 h	56.92 ± 2.65 ^e^	998.20 ± 12.50 ^d^	12.11 ± 0.00 ^a^
		24 h	67.15 ± 1.22 ^bcd^	1127.60 ± 45.80 ^c^	14.74 ± 3.70 ^a^
		48 h	71.90 ± 0.20 ^b^	1239.40 ± 54.10 ^b^	17.37 ± 0.00 ^a^
	Second Fermentation	0 h	65.42 ± 2.45 ^cd^	1239.40 ± 37.40 ^b^	20.00 ± 11.20 ^a^
		6 h	63.83 ± 3.06 ^d^	1124.70 ± 49.90 ^c^	25.26 ± 3.70 ^a^
		12 h	69.88 ± 2.24 ^bc^	1118.80 ± 41.60 ^c^	22.63 ± 7.40 ^a^
	Baking	Kisra	79.39 ± 3.46 ^a^	1433.50 ± 12.50 ^a^	25.26 ± 3.70 ^a^
WC group	First Fermentation	0 h	63.69 ± 1.22 ^c^	1112.90 ± 25.00 ^e^	17.37 ± 7.40 ^a^
		24 h	68.3 ± 2.04 ^bc^	1327.60 ± 20.80 ^bc^	17.37 ± 0.00 ^a^
		48 h	71.04 ± 0.61 ^b^	1357.10 ± 29.10 ^b^	12.11 ± 0.00 ^a^
	Second Fermentation	0 h	70.75 ± 3.06 ^b^	1221.80 ± 20.80 ^d^	17.37 ± 0.00 ^a^
		6 h	67.15 ± 2.45 ^bc^	1177.60 ± 33.30 ^d^	17.37 ± 7.40 ^a^
		12 h	69.31 ± 2.65 ^b^	1280.60 ± 4.20 ^c^	14.74 ± 3.70 ^a^
	Baking	Kisra	85.30 ± 0.00 ^a^	1668.80 ± 12.50 ^a^	27.89 ± 14.90 ^a^

^1^ Control group (kisra naturally fermented without an inoculum), LP group (kisra fermented with *L. plantarum*), LF group (kisra fermented with *L. fermentum*), LB group (kisra fermented with *L. brevis*), WC group (kisra fermented with *W. cibaria*). ^2^ Total phenolic content is expressed as µg of gallic acid equivalent/g of wet samples (Folin–Ciocalteu method). ^3^ Tannin content is expressed as µg of catechin equivalents/g of wet samples (Vanillin–HCl method). ^4^ Data represented mean ± standard deviation from triplicate measurements. Values in the same column of the same group followed by different letters (a–f) are significantly different (*p* < 0.05). The control group shown in this table also served as the natural fermentation group in Table 1.

## Data Availability

Data are contained within this article.

## Supplementary Materials

The following supporting information can be downloaded at: https://www.mdpi.com/article/10.3390/antiox13070844/s1, Table S1: Relative antioxidant properties of kisra bread during natural fermentation; Table S2: Changes in physicochemical and microbial properties during kisra fermentation with or without different lactic acid bacterial species; Table S3: Changes in biogenic amine content during kisra fermentation with or without different lactic acid bacterial species.
