# Supplementary material for: Effects of Lactic Acid Bacteria on Reducing the Formation of Biogenic Amines and Improving the Formation of Antioxidant Compounds in Traditional African Sourdough Flatbread Fermentation
Source: Antioxidants (Basel). 2024 Jul 14;13(7):844. doi: 10.3390/antiox13070844 (PMC11274339; doi:10.3390/antiox13070844)
Supplement: Supplementary file 1 [file antioxidants-13-00844-s001.zip › 3.0-BA in kisra bread-Antioxidants-proofreading-Sup.Tables.pdf]

Table S1. Relative antioxidant properties of kisra bread during natural fermentation.

Processing stages	Antioxidant parameters [1]		
	DPPH (%)	TPC (%)	Tannin (%)
First fermentation			
0 h	100.00	100.00	100.00
24 h	87.70	103.00	76.80
48 h	78.80	99.24	76.80
Second fermentation			
0 h	80.20	102.30	76.80
6 h	83.20	105.30	88.40
12 h	76.90	95.50	123.20
Baking			
kisra bread	118.07	118.32	146.50

[1] Relative antioxidant properties are represented as the percentage with respect to the corresponding 0 hour of fermentation.

Table S2. Changes in physicochemical and microbial properties during kisra fermentation with or without different lactic acid bacterial species.

Groups [1]	Physicochemical and microbial properties	Processing stages						
		**First fermentation**			**Second fermentation**			**Baking**
		**0 h**	**24 h**	**48 h**	**0 h**	**6 h**	**12 h**	**Kisra bread**
Control group (Natural fermentation group)	LAB count (log CFU/g)	8.9±0.13^{2,a}	9.12±0.28^{a}	9.12±0.07^{a}	8.63±0.24^{a}	8.60±0.85^{a}	8.95±0.19^{a}	ND ^{3,b}
	pH	6.74±0.02^{a}	3.68±0.25^{c}	3.23±0.11^{d}	4.49±0.04^{b}	3.72±0.30^{c}	3.53±0.27^{cd}	3.31±0.01^{cd}
	a_w	0.992±0.0004^{a}	0.988±0.001^{ab}	0.986±0.001^{bc}	0.989±0.001^{ab}	0.958±0.04^{abc}	0.985±0.003^{bc}	0.982±0.01^{c}
	TTA **(%)** [4]	0.146±0.002^{c}	0.863±0.10^{b}	1.415±0.40^{a}	0.468±0.05^{bc}	0.62±0.16^{b}	0.746±0.16^{b}	1.635±0.01^{a}
**LP group**	LAB count (log CFU/g)	8.28±0.95^{c}	9.50±0.03^{a}	9.31±0.08^{a}	8.42±0.05^{ba}	9.14±0.09^{ab}	9.23±0.07^{ab}	ND ^{d}
	pH	6.74±0.004^{a}	3.32±0.02^{e}	3.14±0.02^{f}	5.36±0.01^{b}	3.72±0.01^{c}	3.42±0.004^{d}	3.34±0.04^{e}
	a_w	0.992±0.0004^{a}	0.99±0.001^{a}	0.992±0.001^{a}	0.988±0.002^{ab}	0.988±0.001^{ab}	0.986±0.001^{ab}	0.982±0.01^{b}
	TTA **(%)**	0.108±0.02^{g}	0.882±0.02^{c}	1.134±0.01^{b}	0.317±0.002^{f}	0.498±0.004^{e}	0.678±0.02^{d}	1.325±0.05^{a}
**LF group**	LAB count (log CFU/g)	7.64±0.70^{c}	9.45±0.03^{a}	9.31±0.01^{a}	8.48±0.10^{b}	9.09±0.004^{ab}	9.52±0.27^{a}	ND ^{d}
	pH	6.76±0.04^{a}	3.31±0.03^{e}	3.19±0.01^{f}	5.61±0.01^{b}	3.84±0.06^{c}	3.46±0.03^{d}	3.34±0.014^{e}
	a_w	0.992±0.002^{a}	0.990±0.001^{ab}	0.992±0.001^{a}	0.988±0.001^{ab}	0.989±0.001^{ab}	0.985±0.001^{bc}	0.982±0.01^{c}
	TTA **(%)**	0.095±0.01^{e}	0.806±0.01^{bc}	0.959±0.04^{bc}	0.236±0.03^{de}	0.486±0.01^{cd}	0.644±0.002^{bc}	1.47±0.39^{a}
**LB group**	LAB count (log CFU/g)	8.30±1.20^{b}	9.50±0.10^{a}	9.32±0.01^{ab}	8.37±0.24^{b}	9.05±0.08^{ab}	9.23±0.06^{ab}	ND ^{c}
	pH	6.75±0.01^{a}	3.30±0.02^{e}	3.15±0.02^{f}	5.63±0.06^{b}	3.88±0.004^{c}	3.46±0.01^{d}	3.34±0.02^{e}
	a_w	0.993±0.0004^{a}	0.991±0.001^{b}	0.991±0.001^{b}	0.990±0.001^{b}	0.990±0.001^{b}	0.984±0.0004^{c}	0.983±0.0004^{c}
	TTA **(%)**	0.089±0.002^{g}	0.893±0.05^{c}	1.016±0.002^{b}	0.27±0.004^{f}	0.429±0.01^{e}	0.681±0.03^{d}	1.439±0.05^{a}
**WC group**	LAB count (log CFU/g)	7.48±0.33^{b}	9.10±0.10^{a}	7.44±0.06^{bc}	7.10±0.08^{c}	9.15±0.11^{a}	9.23±0.06^{a}	ND ^{d}
	pH	6.77±0.01^{a}	3.77±0.01^{d}	3.75±0.03^{d}	5.99±0.06^{b}	4.25±0.12^{c}	3.85±0.10^{d}	3.50±0.12^{e}
	a_w	0.993±0.002^{a}	0.993±0.001^{b}	0.993±0.002^{b}	0.991±0.0004^{b}	0.996±0.001^{b}	0.986±0.0004^{c}	0.985±0.0004^{c}
	TTA **(%)**	0.09±0.004^{b}	0.545±0.011^{b}	0.569±0.02^{b}	0.303±0.05^{b}	0.434±0.08^{b}	0.557±0.05^{b}	1.143±0.50^{b}

[1] Control group (kisra naturally fermented without an inoculum; this group also served as the natural fermentation group in Figure 2), LP group (kisra fermented with *L. plantarum*), LF group (kisra fermented with *L. fermentum*), LB group (kisra fermented with *L. brevis*), WC group (kisra fermented with *W. cibaria*).
[2] Data represented as mean±standard deviation from triplicate measurements. Values in the same row of the same group followed by different letters (a-g) are significantly different ($p < 0.05$).
[3] ND: not detected.
[4] Total titratable acidity expressed as lactic acid percentage.

Table S3. Changes in biogenic amine content during kisra fermentation with or without different lactic acid bacterial species.

Groups [1]	Processing stages		Biogenic amine content (mg/kg) [2]							
			TRP	PHE	PUT	CAD	HIS	TYR	SPD	SPM
Control group (Natural fermentation group)	First Fermentation	0 h	ND [3]	ND	3.97±0.06[4]^{e}	ND^{e}	ND^{e}	ND^{f}	5.86±0.20^{c}	4.14±0.08^{b}
		24 h	ND	ND	7.05±0.03^{c}	51.58±1.19^{b}	6.64±0.13^{c}	3.67±0.04^{d}	6.42±0.24^{b}	3.83±0.02^{d}
		48 h	ND	ND	26.53±0.27^{a}	69.04±0.91^{a}	3.5±0.08^{d}	15.52±0.07^{a}	6.42±0.01^{b}	3.97±0.07^{c}
	Second Fermentation	0 h	ND	ND	4.19±0.06^{e}	5.60±0.05^{d}	ND^{e}	ND^{f}	4.56±0.05^{e}	3.10±0.01^{e}
		6 h	ND	ND	5.63±0.08^{d}	4.95±0.08^{d}	ND^{e}	2.94±0.08^{e}	5.68±0.08^{c}	3.85±0.02^{d}
		12 h	ND	ND	15.08±0.16^{b}	7.38±0.22^{c}	10.92±0.55^{b}	10.26±0.51^{c}	5.18±0.20^{d}	3.90±0.04^{cd}
	Baking	kisra	ND	ND	26.83±0.72^{a}	6.11±0.07^{cd}	14.76±0.61^{a}	13.73±0.04^{b}	7.72±0.05^{a}	5.04±0.04^{a}
LP group	First Fermentation	0 h	ND	ND	6.55±0.04^{bc}	ND	ND	ND	4.71±0.15^{a}	6.95±0.22^{cd}
		24 h	ND	ND	6.80±0.06^{ab}	ND	ND	ND	3.58±0.08^{c}	6.59±0.10^{d}
		48 h	ND	ND	6.40±0.02^{c}	ND	ND	ND	3.18±0.08^{d}	7.95±0.35^{b}
	Second Fermentation	0 h	ND	ND	6.99±0.33^{a}	ND	ND	ND	4.80±0.09^{a}	8.27±0.24^{b}
		6 h	ND	ND	4.54±0.01^{e}	ND	ND	ND	2.61±0.04^{e}	7.23±0.01^{c}
		12 h	ND	ND	5.72±0.01^{d}	ND	ND	ND	3.78±0.03^{b}	7.24±0.02^{c}
	Baking	kisra	ND	ND	5.99±0.19^{d}	ND	ND	ND	4.79±0.001^{a}	10.54±0.08^{a}
LF group	First Fermentation	0 h	ND	ND	6.58±0.30^{a}	ND	ND	ND	4.41±0.08^{b}	6.97±0.10^{d}
		24 h	ND	ND	5.11±0.08^{c}	ND	ND	ND	3.83±0.04^{c}	6.93±0.03^{d}
		48 h	ND	ND	6.48±0.08^{a}	ND	ND	ND	3.42±0.004^{d}	8.30±0.04^{bc}
	Second Fermentation	0 h	ND	ND	6.10±0.09^{b}	ND	ND	ND	3.97±0.01^{c}	8.10±0.02^{c}
		6 h	ND	ND	5.87±0.03^{b}	ND	ND	ND	3.26±0.02^{e}	6.48±0.02^{e}
		12 h	ND	ND	3.98±0.05^{e}	ND	ND	ND	2.91±0.06^{f}	8.39±0.09^{b}
	Baking	kisra	ND	ND	4.63±0.07^{d}	ND	ND	ND	5.98±0.11^{a}	12.68±0.25^{a}
LB group	First Fermentation	0 h	ND	ND	6.95±0.31^{a}	ND	ND	ND	4.48±0.04^{b}	7.50±0.08^{e}
		24 h	ND	ND	6.40±0.05^{b}	ND	ND	ND	3.49±0.07^{d}	7.29±0.03^{f}
		48 h	ND	ND	6.17±0.01^{b}	ND	ND	ND	3.49±0.01^{d}	8.20±0.07^{d}
	Second Fermentation	0 h	ND	ND	6.15±0.07^{b}	ND	ND	ND	4.78±0.03^{a}	8.24±0.02^{d}
		6 h	ND	ND	5.11±0.11^{c}	ND	ND	ND	3.00±0.02^{e}	8.70±0.06^{c}
		12 h	ND	ND	5.03±0.003^{c}	ND	ND	ND	3.05±0.06^{e}	9.13±0.02^{a}
	Baking	kisra	ND	ND	4.87±0.19^{c}	ND	ND	ND	3.75±0.11^{c}	8.90±0.01^{b}
WC group	First Fermentation	0 h	ND	ND	7.04±0.32^{a}	ND	ND	ND	3.96±0.002^{g}	8.26±0.15^{b}
		24 h	ND	ND	7.10±0.05^{a}	ND	ND	ND	4.60±0.05^{f}	6.66±0.03^{d}
		48 h	ND	ND	6.94±0.11^{a}	ND	ND	ND	4.86±0.04^{e}	6.71±0.12^{d}
	Second Fermentation	0 h	ND	ND	6.49±0.16^{b}	ND	ND	ND	5.39±0.10^{c}	8.00±0.05^{b}
		6 h	ND	ND	6.08±0.07^{c}	ND	ND	ND	4.99±0.03^{d}	7.39±0.13^{c}
		12 h	ND	ND	5.67±0.02^{d}	ND	ND	ND	6.21±0.04^{b}	8.51±0.03^{b}
	Baking	kisra	ND	ND	5.00±0.001^{e}	ND	ND	ND	6.72±0.01^{a}	10.38±0.52^{a}

[1] Control group (kisra naturally fermented without an inoculum; this group also served as the natural fermentation group in Figure 3), LP group (kisra fermented with *L. plantarum*), LF group (kisra fermented with *L. fermentum*), LB group (kisra fermented with *L. brevis*), WC group (kisra fermented with *W. cibaria*).

[2] TRP: tryptamine; PHE: β-phenylethylamine; PUT: putrescine; CAD: cadaverine; HIS: histamine; TYR: tyramine; SPD: spermidine; SPM: spermine.

[3] ND: not detected.

[4] Data represented as mean±standard deviation from triplicate measurements. Values in the same column of the same group followed by different letters (a-g) are significantly different ($p < 0.05$).
